# The Effect of Ovarian Endometriosis on Pregnancy Outcomes in Spontaneous Pregnancies

**DOI:** 10.3390/jcm14103468

**Published:** 2025-05-15

**Authors:** Halis Dogukan Ozkan, Merve Ayas Ozkan, Ahmet Arif Filiz, Muhammed Enes Karakaya, Yaprak Engin-Ustun

**Affiliations:** 1 Department of Obstetrics and Gynecology, Lösante Children and Adults Hospital, Ankara 06830, Turkey; 2 Department of Obstetrics and Gynecology, Division of Perinatology, Ankara Etlik City Hospital, Ankara 06710, Turkey; merveayasozkan@gmail.com (M.A.O.); ahmetarif35.filiz@gmail.com (A.A.F.); 3 Department of Obstetrics and Gynecology, Etlik Zübeyde Hanım Women’s Diseases Training and Research Hospital, Ankara 06010, Turkey; muh.enessss@gmail.com (M.E.K.); ustunyaprak@yahoo.com (Y.E.-U.)

**Keywords:** ovarian endometriosis, pregnancy complications, placenta previa, preterm labor, abortion

## Abstract

**Background:** This study investigates the impact of ovarian endometriosis on pregnancy outcomes. **Methods:** A retrospective analysis was conducted at Etlik Zübeyde Hanım Women’s Diseases Training and Research Hospital between January 2019 and December 2024, including 1127 pregnant women—170 with ovarian endometriosis and 957 healthy controls. Pregnancies achieved via assisted reproductive techniques were excluded. Statistical analyses were performed using appropriate tests, and a *p*-value < 0.05 was considered significant. **Results:** Women with ovarian endometriosis had higher rates of miscarriage (21.8% vs. 7.5%), preterm birth (15.0% vs. 8.8%), and placenta previa (4.7% vs. 0.6%), with adjusted odds ratios (OR) of 3.41, 1.84, and 7.82, respectively. No significant differences were observed in terms of gestational diabetes, hypertensive disorders, fetal growth restriction (FGR), intrahepatic cholestasis of pregnancy (ICP), placental abruption, or preterm premature rupture of membranes (PPROM). Cyst size and bilaterality were not associated with complications. **Conclusions:** Spontaneously conceiving women with ovarian endometriosis are at increased risk for miscarriage, placenta previa, and preterm birth. Prospective randomized studies are warranted to validate these findings.

## 1. Introduction

Endometriosis is a chronic benign condition commonly observed in women of reproductive age. Numerous theories have been proposed regarding its pathogenesis, with the most widely accepted being the theory of retrograde menstruation via the fallopian tubes [1]. In endometriosis, increased inflammatory mediators in the peritoneum cause chronic inflammation. The inflammation induced by ectopic endometrial tissue and the resulting adhesions manifest clinically as chronic pelvic pain, dysmenorrhea, and dyspareunia [2]. Although endometriosis can affect multiple organs, the ovaries are the most frequently involved sites [3].

Increased progesterone levels during pregnancy are considered to alleviate the symptoms of endometriosis [4]. However, several studies have demonstrated that endometriosis is associated with a variety of pregnancy complications [5]. The chronic inflammation inherent to endometriosis is considered to contribute to these complications [6]. In particular, endometriosis has been linked to miscarriage, preterm birth, hypertensive disorders of pregnancy, gestational diabetes, placental invasion abnormalities, fetal growth restriction, and cesarean delivery [6,7,8,9,10,11]. However, there is no established additional follow-up protocol for pregnant women with endometriosis [12,13]. According to the guidelines of the European Society of Human Reproduction and Embryology (ESHRE), although there is currently no recommendation for increased follow-up care for pregnant women with endometriosis, clinicians should remain aware of the potential for complications during pregnancy [14]. Most studies have focused on patients who conceived via assisted reproductive techniques (ARTs). It is known that an ART itself is associated with pregnancy complications [15]. In contrast, investigations into pregnancy complications following spontaneous conception in women with endometriosis are relatively limited. Moreover, few studies specifically address pregnancy outcomes in women with ovarian endometriosis. The aim of this study was to contribute to bridging this gap in relevant literature.

In this study, we specifically selected patients with ovarian endometriosis in order to more clearly evaluate the effects of endometriosis on pregnancy outcomes and to create a more homogeneous study group. Ovarian endometriosis, in addition to being the most common form of endometriosis, can directly affect ovarian reserve and ovulatory function through stromal fibrosis and chronic inflammation, similar to other types of endometriosis [3,16]. In contrast, subtypes such as deep infiltrative endometriosis (DIE) do not have easily detectable ultrasound findings such as ovarian cysts. Diagnosis often requires transvaginal ultrasound, magnetic resonance imaging, and surgical intervention, which can introduce variability and bias in outcome analyses [17]. Therefore, patients with DIE or isolated peritoneal endometriosis were excluded from the study. Researchers Vercellini and Uccella have previously investigated pregnancy outcomes related to endometriosis, incorporating ovarian endometriosis in their analyses [18,19]. However, our study is novel in that it focuses exclusively on women with ovarian endometriosis who conceived spontaneously and includes a larger number of patients, enabling an analysis of outcomes in this specific group. This approach allows for a better understanding of the specific risks associated with ovarian endometriosis without the confounding factors of ART or different subtypes of endometriosis. A retrospective design focusing on this population is justified to eliminate the confounding effects of ART and to better understand the natural progression of pregnancy in the presence of this specific phenotype of endometriosis.

## 2. Materials and Methods

### 2.1. Sample Collection

This retrospective study was designed to compare pregnancy outcomes between patients diagnosed with ovarian endometriosis and those without ovarian endometriosis at the Etlik Zübeyde Hanım Women’s Diseases Training and Research Hospital between January 2019 and December 2024. The Etlik Zübeyde Hanım Women’s Diseases Hospital is one of the most reputable centers in Turkey that specializes in women’s health. This study received ethical approval (decision number 2024/15, dated 9 December 2024) and was conducted in accordance with the Declaration of Helsinki.

Pregnant women aged 18–40 years who delivered at this hospital were included; those diagnosed with ovarian endometriosis comprised the study group, whereas healthy pregnancies formed the control group. Patients considered to be at increased risk for pregnancy complications, including those who conceived via in vitro fertilization (IVF), those with chronic diseases, multiple pregnancies, individuals with a history of smoking, and alcohol dependence, were excluded from this study. In addition, patients with a history of pregnancy complications (e.g., placenta previa/abruptio, preterm birth, fetal growth restriction, premature rupture of membranes, intrahepatic cholestasis, gestational hypertension, or gestational diabetes) in previous pregnancies were also excluded from the study. It is known that these complications carry a risk of recurrence in subsequent pregnancies [20,21]. Therefore, this exclusion was made to minimize the impact of risk factors unrelated to endometriosis on the study results. All maternal data were obtained from the hospital’s information system. In total, 1127 patients meeting the inclusion criteria were enrolled in the study, comprising 957 healthy pregnant women (control group) and 170 pregnant women diagnosed with ovarian endometriosis (endometriosis group). Ovarian endometriosis was diagnosed histopathologically in patients who underwent surgery, and via ultrasound and magnetic resonance imaging (MRI) in those who did not [22]. Ultrasound examinations at our hospital are performed by a team specializing in endometriosis. To confirm the diagnosis of ovarian masses, only patients whose repeated ultrasound examinations consistently showed an endometrioma were included in the study. Patients with suspected or confirmed deep infiltrating endometriosis (DIE) or isolated peritoneal endometriosis were excluded to ensure a homogeneous study population focused solely on ovarian endometriosis.

Maternal characteristics recorded included age, gravidity, parity, history of miscarriage, history of vaginal or cesarean delivery, pre-pregnancy body mass index (BMI), dimensions of ovarian endometriosis, and bilaterality. Maternal outcomes included mode of delivery, gestational age at birth, and pregnancy complications (miscarriage, preterm birth, PPROM, hypertensive disorders of pregnancy, gestational diabetes, ICP, FGR, placental abruption, and placenta previa). Preterm birth was defined as delivery before the 37th week of gestation, and PPROM as the rupture of fetal membranes before the 37th week of gestation [23,24]. The diagnoses of pregnancy-induced hypertension, gestational diabetes mellitus, and ICP were made according to the American College of Obstetricians and Gynecologists guidelines [25,26,27]. FGR was diagnosed based on the Delphi criteria [28]. Placenta previa was defined as the placental extension to or complete coverage of the internal cervical os [29].

### 2.2. Statistical Analysis

Statistical analyses were performed using SPSS version 22.0 (IBM Corporation, Armonk, NY, USA). The Kolmogorov–Smirnov test was used to analyze conformity to normal distribution. Descriptive statistics of continuous variables are shown as “mean ± standard deviation” for those with normal distribution and as “median (interquartile range)” for those without normal distribution. Categorical variables were compared using the chi-squared test or Fisher’s exact test. Continuous variables that were and were not normally distributed were compared using the independent sample *t*-test and the Mann–Whitney U test, respectively. Statistical significance was defined as a *p*-value less than 0.05.

## 3. Results

A total of 1127 patients were included in the study. As shown in Table 1, no statistically significant differences were found between the endometriosis and control groups in terms of age and BMI. The mean age was 27.5 ± 4.4 years in the control group and 26.1 ± 4.2 years in the endometriosis group. The mean BMI was 26.1 ± 3.7 kg/m^2^ in the control group and 24.6 ± 3.5 kg/m^2^ in the endometriosis group. Although both groups had a median gravidity of 2, the *p*-value was <0.001, indicating a statistically significant difference. Similarly, median parity was 1 in both groups; however, the *p*-value was 0.001, showing a statistically significant difference between the groups. In addition, 65.7% of participants in the control group were nulliparous, compared with 54.1% of participants in the endometriosis group (*p* = 0.004). Although the differences in age and BMI between the groups were not statistically significant, it is worth noting that the endometriosis group tended to be slightly younger and had a lower mean BMI compared to the control group.

As shown in Table 2, the rates of preterm birth, placenta previa, and miscarriage were significantly higher in the endometriosis group. The number of preterm births was 20 (15%) in the endometriosis group, compared with 77 (8.8%) in the control group (*p* = 0.022). The number of patients diagnosed with placenta previa was 8 (4.7%) in the endometriosis group versus 6 (0.6%) in the control group (*p* < 0.001). This difference indicated a significantly increased risk of placenta previa in the presence of endometriosis. The miscarriage rates were 21.8% (*n* = 37) in the endometriosis group and 7.5% (*n* = 72) in the control group (*p* < 0.001). The adjusted OR for preterm birth, placenta previa, and miscarriage were calculated as 1.84 (95% confidence interval (CI), 1.08–3.19), 7.82 (95% CI, 2.68–22.85), and 3.41 (95% CI, 2.21–5.29), respectively. With respect to the other complications, no statistically significant differences were found between the groups for PPROM, gestational diabetes mellitus, fetal growth restriction, gestational hypertension, cholestasis, or the requirement for cesarean delivery.

**Figure 1:**
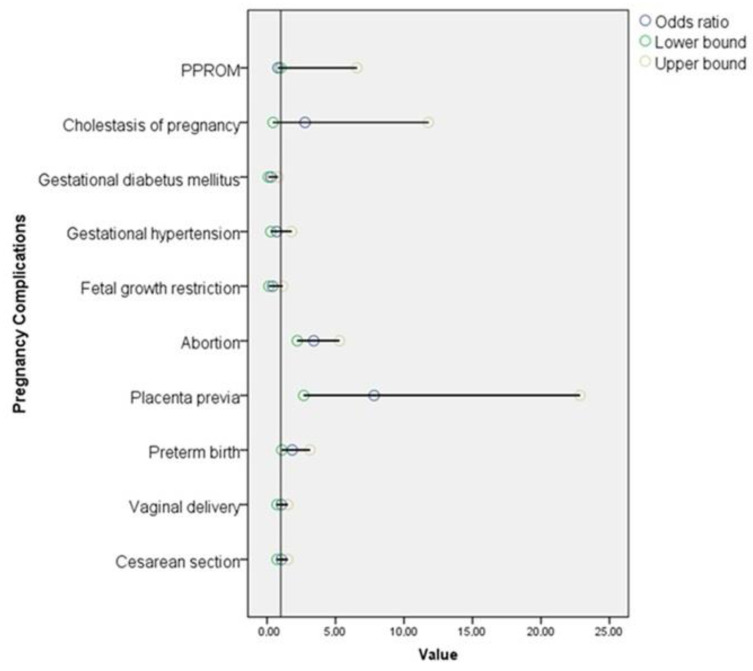
Forest plot presenting the odds ratios (OR) and 95% confidence intervals (CI) for pregnancy complications in women with ovarian endometriosis compared to healthy controls. Each line represents a specific complication, with the dot indicating the odds ratio, and the horizontal line representing the lower and upper bounds of the 95% confidence interval. An odds ratio greater than 1 indicates increased risk associated with endometriosis.

Figure 1 illustrates the odds ratios and 95% confidence intervals for pregnancy complications in women with ovarian endometriosis compared to healthy controls. Among the examined complications, the risk of miscarriage, placenta previa, and preterm birth was significantly higher in the endometriosis group. Other outcomes such as PPROM, gestational diabetes, and cesarean delivery did not show statistically significant differences.

Table 3 demonstrates the relationship between cyst diameter and bilaterality with pregnancy complications in the endometriosis group. The mean cyst diameter was 51.62 ± 27.72 mm in patients with placenta previa and 54.02 ± 24.81 mm in patients without placenta previa; 25% were bilateral. The corresponding *p*-values were 0.776 and 0.353, respectively, indicating no statistically significant differences. The mean cyst diameter was 57.4 ± 25.62 mm in patients with preterm birth, compared to 54.47 ± 24.62 mm in patients without preterm birth; however, this difference was not statistically significant (*p* = 0.628). The presence of bilateral cysts was also not significant among patients with preterm birth (*p* = 0.522). Furthermore, when comparing patients with and without miscarriage, no statistically significant association was found between cyst diameter or bilaterality (*p* = 0.407 and *p* = 0.734, respectively). Similarly, no significant associations were observed between cyst diameter or bilaterality and the other pregnancy complications.

## 4. Discussion

The aim of this study was to evaluate whether women with ovarian endometriosis experience increased pregnancy complications compared to healthy pregnancies. Ovarian endometriosis is thought to affect not only through local ovarian effects but also through systemic inflammatory pathways [30]. Pro-inflammatory and immunoregulatory cytokines are markedly increased in the peritoneal fluid of patients with endometrioma [31,32]. These factors may affect implantation and placentation by impairing endometrial receptivity, decidualization, and trophoblast invasion [33,34,35]. Furthermore, systemic inflammation may contribute to endothelial dysfunction and abnormal vascular remodeling, creating a potential link with pregnancy complications [36]. Similarly, tissue damage due to chronic inflammation, the formation of adhesions in the pelvis, and pathological contractions due to inflammatory conditions in the endometrium may also be associated with obstetric complications [12]. Although previous reports have addressed this issue, those evaluating isolated ovarian endometriosis included a small number of patients [18,19]. In the present study, pregnant women diagnosed with endometrioma (*n* = 170) exhibited increased risks of miscarriage, placenta previa, and preterm birth compared to healthy pregnant women (*n* = 957). No association was found between endometrioma and the other evaluated pregnancy complications. Furthermore, the size of the ovarian cysts and their bilaterality had neither positive nor negative effects on pregnancy complications.

Large meta-analyses and cohort studies have shown that the risk of placenta previa is increased in women with endometriosis [37,38,39]. However, most of these studies were conducted in pregnancies conceived via ART, which are themselves associated with placenta previa [15]. This may have influenced the observed association between endometriosis and placenta previa. Moreover, several previous studies have been performed without accounting for the localization of endometriosis. In a study by Vercellini et al., spontaneously conceived pregnancies in women with rectovaginal, peritoneal, and ovarian endometriosis were evaluated; among the 100 women with ovarian endometriosis included in the study, none presented with placenta previa, whereas women with rectovaginal endometriosis had an approximately tenfold increased risk [19]. Similarly, Ucella et al. evaluated 64 women with ovarian endometriosis who conceived spontaneously based on the localization of endometriosis and reported that none of the patients showed placenta previa [18]. In contrast, the present study, which included only 170 patients with spontaneous pregnancies and ovarian endometriosis, demonstrated that the risk of placenta previa was 7.8 times higher compared to in the healthy control group (OR: 7.82, 95% CI: 2.68–22.85). It is known that endometriosis increases angiogenesis in the endometrium in association with chronic inflammation [36]. Similarly, inflammation and the associated increase in angiogenesis have been implicated in the pathology of placenta previa [40,41]. This suggests that the increased risk of placenta previa in endometriosis may be related to inflammatory processes. In addition, abnormal uterine contractility in endometriosis, which may affect blastocyst implantation, has also been proposed as a potential cause of placenta previa [42].

Preterm birth is a serious condition influenced by multiple factors, resulting in neonatal morbidity and mortality [23]. The achievement of pregnancies through ART has been identified as one of the factors that increases the risk of preterm birth [43]. Fernando et al. demonstrated that, in ART pregnancies with ovarian endometriosis, the risk of preterm birth was twice as high compared to natural conception [44]. However, it is difficult to determine whether this increase is attributable to endometriosis itself or to the ART procedure. It is known that inflammatory cytokines are increased in endometriosis. Some of these cytokines include interleukin-6, interleukin-1β, and TNF-α. These cytokines have also been shown to play an important role in the mechanism of preterm birth, which makes the increased incidence of preterm birth in patients with endometriosis plausible [12]. In contrast to the mechanism described in the literature, a study in which women with endometrioma who became pregnant spontaneously were examined found that the risk of preterm birth was lower than in healthy controls [45]. In a large meta-analysis, the risk of preterm birth was found to be increased in ART pregnancies; however, the authors noted that data were inadequate to compare between non-ART pregnancies or among different subtypes of endometriosis, as most studies did not specify the type [39]. In a prospective study in which endometriosis was diagnosed laparoscopically, 49 women with endometriosis who became pregnant through ART (IVF or artificial insemination) were compared with 59 healthy controls, and no significant difference was found between the groups in terms of pregnancy outcome [13]. This suggests that the association between endometriosis and pregnancy complications may differ depending on the mode of conception and the study population. Another study comparing pregnancy outcomes in spontaneously conceived pregnancies in women with endometriosis also reported an increased risk of preterm birth; however, the study did not provide further details on pregnancy outcomes in various endometriosis subgroups [46]. In the present study, evaluating 170 pregnant women with ovarian endometriosis who conceived spontaneously, the risk of preterm birth was found to be 1.84 times higher (OR: 1.84 95% CI: 1.08–3.19); however, cyst dimensions were not associated with this condition.

Endometriosis-induced inflammatory changes and the consequent structural alterations in intra-abdominal organs increase the risk of pregnancy complications [6]. Endometriosis is believed to possibly result in miscarriages through mechanisms of impaired uterine contractility and disrupted endometrial receptivity [42]. In a meta-analysis by Zullo et al., the risk of miscarriage was found to be elevated in pregnancies complicated by endometriosis [39]. Another meta-analysis conducted in 2020 reported that pregnant women with endometriosis have a higher risk of miscarriage compared to those without the condition [47]. Similarly, Farland et al. observed an increased miscarriage risk in studies of spontaneously conceived pregnancies in women with endometriosis [5]. In line with these findings, the present study also demonstrated an increased risk of miscarriage (OR: 3.41 95% CI: 2.21–5.29). Although Vercellini et al. reported a higher spontaneous miscarriage risk in pregnancies with ovarian endometrioma, they suggested that this might be related to the higher mean age of that group [19].

Although previous studies attributed the increased risk of miscarriage to the older maternal age in the endometriosis group [19], in our study, the average age of the endometriosis group was actually lower, but this difference was at the threshold of statistical significance (*p* = 0.053). It is known in the literature that the prevalence of endometriosis increases with age (peaking between 35 and 45 years) [48] and that pregnancy complications also increase with advanced age (especially over 35 years) [49]. In our study, the average age of the endometriosis group was 26.1, while the average age of the control group was 27.5. Although BMI did not differ significantly between the groups, the control group had a slightly higher mean BMI. Despite the higher age and BMI of the control group, no significant difference was observed that would increase pregnancy complications. Moreover, despite the younger age of the endometriosis group, certain pregnancy complications were more common in this cohort (placenta previa, preterm birth, and miscarriage), which may reflect the specific pathophysiological impact of endometriosis rather than metabolic risk factors.

No significant association was observed between endometrioma and hypertensive disorders of pregnancy, gestational diabetes, ICP, FGR, placental abruption, and PPROM in this study. Similar to the present study, Ucella et al. found no significant differences between women with endometrioma and controls regarding hypertensive disorders, gestational diabetes mellitus, or FGR [18]. Conversely, other studies have reported associations between endometriosis and PPROM, hypertensive disorders, gestational diabetes mellitus, FGR [11], placental abruption [37,38], and ICP [50]. The literature highlights the association between endometriosis and systemic inflammation, resulting in endothelial dysfunction and abnormal vascular remodeling [36]. Therefore, an increase in pregnancy complications such as hypertensive disorders of pregnancy and fetal growth restriction in endometriosis may be expected. The absence of a significant association between these complications and endometriosis in our study, despite being consistent with some studies in the literature, contradicts the proposed mechanism; this discrepancy may be explained by the type of endometriosis (only ovarian endometriosis was included), population characteristics, or sample size limitations. A recent meta-analysis found that unfavorable pregnancy outcomes are more common in primiparous pregnant women with endometriosis than in multiparous pregnant women with endometriosis and that parity may have an impact on unfavorable pregnancy outcomes [51]. However, the quality of the studies included in this meta-analysis was rated as low to very low. It was also highlighted that only hypertensive disorders of pregnancy increased as unfavorable pregnancy outcomes, while there was no significant difference in other pregnancy complications. Although parity was not considered as a primary variable in our study and the median of parity was 1 in both groups, parity was significantly higher in the control group (healthy pregnant women). However, in our study, no significant difference was found between the healthy control group and pregnant women with ovarian endometriosis in terms of hypertensive pregnancy disorders.

This study was limited by its single-center and retrospective design, which may restrict the generalizability of our findings. Although endometrioma was diagnosed by an experienced team using transvaginal ultrasound and MRI and confirmed, through repeated ultrasonography, the absence of pathological confirmation remains a limitation. In our study, pregnant women who had experienced obstetric complications in a previous pregnancy were excluded. This decision was made to prevent pre-existing obstetric risks from influencing the results. However, this limitation may restrict the generalizability of our findings to all pregnant women with endometriosis. Furthermore, we did not stratify the patients according to the stage of endometriosis. Previous studies have shown that the severity of endometriosis can influence the risk of certain pregnancy complications such as pre-eclampsia and preterm labor, especially in advanced stages [52]. Therefore, the lack of staging in our analysis is a further limitation.

The most significant strength of this study is that all pregnancies were spontaneous, thereby excluding potential complications associated with IVF. Furthermore, to the best of our knowledge, there are no larger studies in the literature that deal exclusively with ovarian endometriosis, which is another strength of our study.

## 5. Conclusions

In conclusion, this study demonstrates that women with ovarian endometriosis who conceive spontaneously are at increased risk for miscarriage, placenta previa, and preterm birth. Ovarian endometriosis can adversely affect the normal course of pregnancy. It is imperative that clinicians exercise heightened vigilance and provide appropriate counseling to patients with ovarian endometriosis. Further prospective research, including randomized controlled studies, is necessary to verify these retrospective findings. However, conducting such a study in this patient group presents ethical and practical challenges. Therefore, well-designed prospective observational studies may offer a more feasible alternative. Given the complication risks identified in our study, it may be prudent to exercise caution regarding miscarriage, placenta previa, and preterm birth in women with ovarian endometriosis, even in the absence of clear recommendations in current guidelines. Further studies are needed to evaluate the necessity of special pregnancy monitoring in women with endometriosis.

## Figures and Tables

**Table 1:** Descriptive and comparative analysis of demographic data between the endometriosis and control groups.

Parameter	Control Group *n* = 957 (84.9%)	Ovarian Endometriosis Group *n* = 170 (15.1%)	*p*-Value
**Age (y)**	27.5 ± 4.4	26.1 ± 4.2	0.053 ^a^
**BMI (kg/m^2^)**	26.1 ± 3.5	24.6 ± 3.5	0.563 ^a^
**Gravida**	2 (2)	2 (2)	**<0.001 ^b^**
**Parity**	1 (2)	1 (1)	**0.001 ^b^**
**Nulliparous**	629 (65.7%)	92 (54.1%)	**0.004 ^c^**

Data are expressed as *n* (%), mean ± SD or median (interquartile range) where appropriate. A *p*-value of <0.05 indicates a significant difference and statistically significant *p*-values are in bold. ^a^: Student’s *t*-test, ^b^: Mann–Whitney U, ^c^: Pearson chi-square.

**Table 2:** Comparison of the rate of pregnancy complications in endometriosis and control groups.

Parameter	Control Group *n* = 957 (84.9%)	Ovarian Endometriosis Group *n* = 170 (15.1%)	Odds Ratio (95% CI)	*p*-Value
**PPROM**	7 (0.7%)	1 (0.6%)	0.80 (1–6.57)	1 ^a^
**Abruptio placenta**	3 (0.3%)	0 (0%)	NA	1 ^a^
**Cholestasis of pregnancy**	5 (0.5%)	2 (1.2%)	2.27 (0.44–11.78)	0.282 ^a^
**Gestational diabetes mellitus**	47 (4.9%)	4 (2.4%)	0.48 (0.16–0.87)	0.139 ^a^
**Gestational hypertension**	40 (4.2%)	5 (2.9%)	0.70 (0.27–1.79)	0.447 ^b^
**Fetal growth restriction**	54 (5.6%)	4 (2.4%)	0.40 (0.14–1.13)	0.073 ^a^
**Abortion**	72 (7.5%)	37 (21.8%)	**3.41 (2.21–5.29)**	**<0.001 ^b^**
**Placenta previa**	6 (0.6%)	8 (4.7%)	**7.82 (2.68–22.85)**	**<0.001 ^b^**
**Preterm birth**	77 (8.8%)	20 (15%)	**1.84 (1.08–3.13)**	**0.022 ^b^**
**Vaginal delivery**	437 (49.4%)	64 (48.1%)	1.05 (0.73–1.51)	0.777 ^b^
**Cesarean section**	447 (50.6%)	69 (51.9%)	1.05 (0.73–1.51)	0.777 ^b^

More than one pregnancy complication can occur simultaneously in the same patient. When calculating the rates associated with pregnancy complications, each complication was calculated separately. A *p*-value of <0.05 indicates a significant difference and statistically significant *p*-values are in bold. ^a^: Fisher’s exact test, ^b^: Pearson chi-square, CI: confidence interval, NA: Not applicable.

**Table 3:** Comparison of cyst diameter and bilaterality in the ovarian endometriosis group according to presence or absence of pregnancy complications.

Parameter		Complication Absent	Complication Present	*p*-Value
**PPROM**	Diameter of cyst	54.2 ± 24.8	32	0.375 ^a^
	Bilaterality	26 (15.4%)	0 (0%)	1 ^b^
**Abruptio placenta**	Diameter of cyst	54.07 ± 24.8	NA	NA
	Bilaterality	26 (15.3%)	NA	NA
**Cholestasis of pregnancy**	Diameter of cyst	54.17 ± 24.9	45.75 ± 8.1	0.363 ^a^
	Bilaterality	26 (15.5%)	0 (0%)	1 ^b^
**Gestational diabetus mellitus**	Diameter of cyst	54.09 ± 25.1	53.37 ± 13.3	0.923 ^a^
	Bilaterality	25 (15.1%)	1 (25%)	0.489 ^b^
**Gestational hypertension**	Diameter of cyst	54.14 ± 24.45	51.9 ± 40	0.843 ^a^
	Bilaterality	24 (14.5%)	2 (40%)	0.168 ^b^
**Fetal growth restriction**	Diameter of cyst	54.41 ± 24.95	40 ± 18.01	0.253 ^a^
	Bilaterality	26 (15.7%)	0 (0%)	1 ^b^
**Miscarriage**	Diameter of cyst	54.91 ± 24.69	51.06 ± 25.59	0.407 ^a^
	Bilaterality	21 (15.8%)	5 (13.5%)	0.734 ^c^
**Placenta previa**	Diameter of cyst	54.02 ± 24.81	51.62 ± 27.72	0.776 ^a^
	Bilaterality	24 (14.8%)	2 (25%)	0.353 ^b^
**Pregnancy complications**	Diameter of cyst	52.46 ± 25.31	55.38 ± 24.56	0.447 ^a^
	Bilaterality	9 (11.8%)	17 (18.1%)	0.261 ^b^
**Preterm birth**	Diameter of cyst	54.47 ± 24.62	57.4 ± 25.62	0.628 ^a^
	Bilaterality	17 (15%)	4 (20%)	0.522 ^b^
**Cesarean section**	Diameter of cyst	52.04 ± 21.12	57.57 ± 27.49	0.198 ^a^
	Bilaterality	10 (15.6%)	11 (15.9%)	0.960 ^c^

NA: Not applicable, ^a^: Student’s *t*-test, ^b^: Fisher’s exact test, ^c^: Pearson chi-square.

## Data Availability

If requested, data can be shared by the corresponding author with patient names and IDs anonymized.

## Abbreviations

The following abbreviations are used in this manuscript:

ART	Assisted reproductive techniques
BMI	Body mass index
IVF	In vitro fertilization
PPROM	Preterm premature rupture of membranes
ICP	Intrahepatic cholestasis of pregnancy
FGR	Fetal growth restriction
OR	Odds ratio
CI	Confidence interval
